# Methyl-CpG-binding protein 2 mediates overlapping mechanisms across brain disorders

**DOI:** 10.1038/s41598-020-79268-0

**Published:** 2020-12-17

**Authors:** Snow Bach, Niamh M. Ryan, Paolo Guasoni, Aiden P. Corvin, Rania A. El-Nemr, Danyal Khan, Albert Sanfeliu, Daniela Tropea

**Affiliations:** 1 School of Mathematical Sciences, Dublin City University, Glasnevin, Dublin 9, D09 W6Y4 Ireland; 2 Neuropsychiatric Genetics, Department of Psychiatry, Trinity College Dublin, School of Medicine, Trinity Translational Medicine Institute, Trinity Center for Health Sciences, St James Hospital, Dublin 8, Dublin, Ireland; 3 Department of Mathematics and Statistics, Boston University, 111 Cummington Street, Boston, MA 02215 USA; 4 School of Medicine, Trinity College Dublin, Dublin, Ireland; 5 Trinity College Institute of Neuroscience, Trinity College Dublin, Lloyd Building, Dublin 2, Dublin, Ireland; 6 FutureNeuro, The SFI Research Centre for Chronic and Rare Neurological Diseases, Dublin, Ireland

**Keywords:** Diseases of the nervous system, Epigenetics in the nervous system, Neuroscience, Diseases

## Abstract

*MECP2* and its product, Methyl-CpG binding protein 2 (MeCP2), are mostly known for their association to Rett Syndrome (RTT), a rare neurodevelopmental disorder. Additional evidence suggests that *MECP2* may underlie other neuropsychiatric and neurological conditions, and perhaps modulate common presentations and pathophysiology across disorders. To clarify the mechanisms of these interactions, we develop a method that uses the binding properties of MeCP2 to identify its targets, and in particular, the genes recognized by MeCP2 and associated to several neurological and neuropsychiatric disorders. Analysing mechanisms and pathways modulated by these genes, we find that they are involved in three main processes: neuronal transmission, immuno-reactivity, and development. Also, while the nervous system is the most relevant in the pathophysiology of the disorders, additional systems may contribute to MeCP2 action through its target genes. We tested our results with transcriptome analysis on *Mecp2*-null models and cells derived from a patient with RTT, confirming that the genes identified by our procedure are directly modulated by MeCP2. Thus, MeCP2 may modulate similar mechanisms in different pathologies, suggesting that treatments for one condition may be effective for related disorders.

## Introduction

MeCP2 is a protein that controls gene expression levels through direct and indirect mechanisms. Generally, it acts as repressor by binding methylated CpG dinucleotides to modify chromatin. However, it may also work as an activator by interacting with specific co-factors such as CREB1. One of the MeCP2′s target is Brain-derived Neurotrophic Factor (BDNF), a neurotrophin involved in brain development and function^1^. BDNF-related mechanisms are dysregulated as a result of *MECP2* mutations, and altered BDNF expression has been detected in several disorders, including neurodevelopmental disorders, depression, and anxiety^2,3^.

Initially identified as an oncogene, the *MECP2* gene is now mostly associated with Rett Syndrome (RTT): a progressive X-linked neurological disorder that primarily affects females. However, *RTT-*similar phenotypes have been identified across different syndromes^4–8^, and *MECP2* is involved in several other neuropsychiatric and neurological conditions^9^, with its dysregulation having functional consequences^10^.

In this study, we develop a procedure to identify potential MeCP2 binding sites over false positives, and we apply this procedure to selected gene-sets derived from several genetic studies on neuropathologies: autism^11^, attention deficit hyperactivity disorder (ADHD)^12^, major depressive disorder (MDD)^13^, bipolar disorder (BIP)^14^ ,anorexia^15^, epilepsy^16^, Alzheimer’s disease (AD)^17^, Parkinson’s Disease (PD)^18^, Huntington’s disease (HTT)^19^, amyotrophic lateral sclerosis (ALS)^20^, multiple sclerosis (MS)^21^, and schizophrenia (SCZ)^22^.

Using this approach, we show that MeCP2 binds the promoters of genes associated with brain disorders more often than expected by chance. Additional single nucleotide polymorphism (SNP) analysis confirms that mutations in *MECP2* are present in several of the investigated conditions, suggesting that some biological mechanisms operating in different brain disorders are modulated by MeCP2.

In order to identify these mechanisms, we use the candidate MeCP2 target genes from our analysis, to investigate tissue expression profiles and carry out enrichment and network analysis controlling for false positives. Transcriptome analysis in mouse mutants of *Mecp2* and in induced pluripotent stem cells (iPSC) derived from a patient with RTT confirms that the majority of genes identified with our methods are differentially expressed compared to controls.

Our results propose unexpected connections between MeCP2 and different brain pathologies, and suggest that common molecular mechanisms active across several brain disorders are modulated by MeCP2.

## Methods

### Establishing MeCP2 binding sites

**Figure 1:**
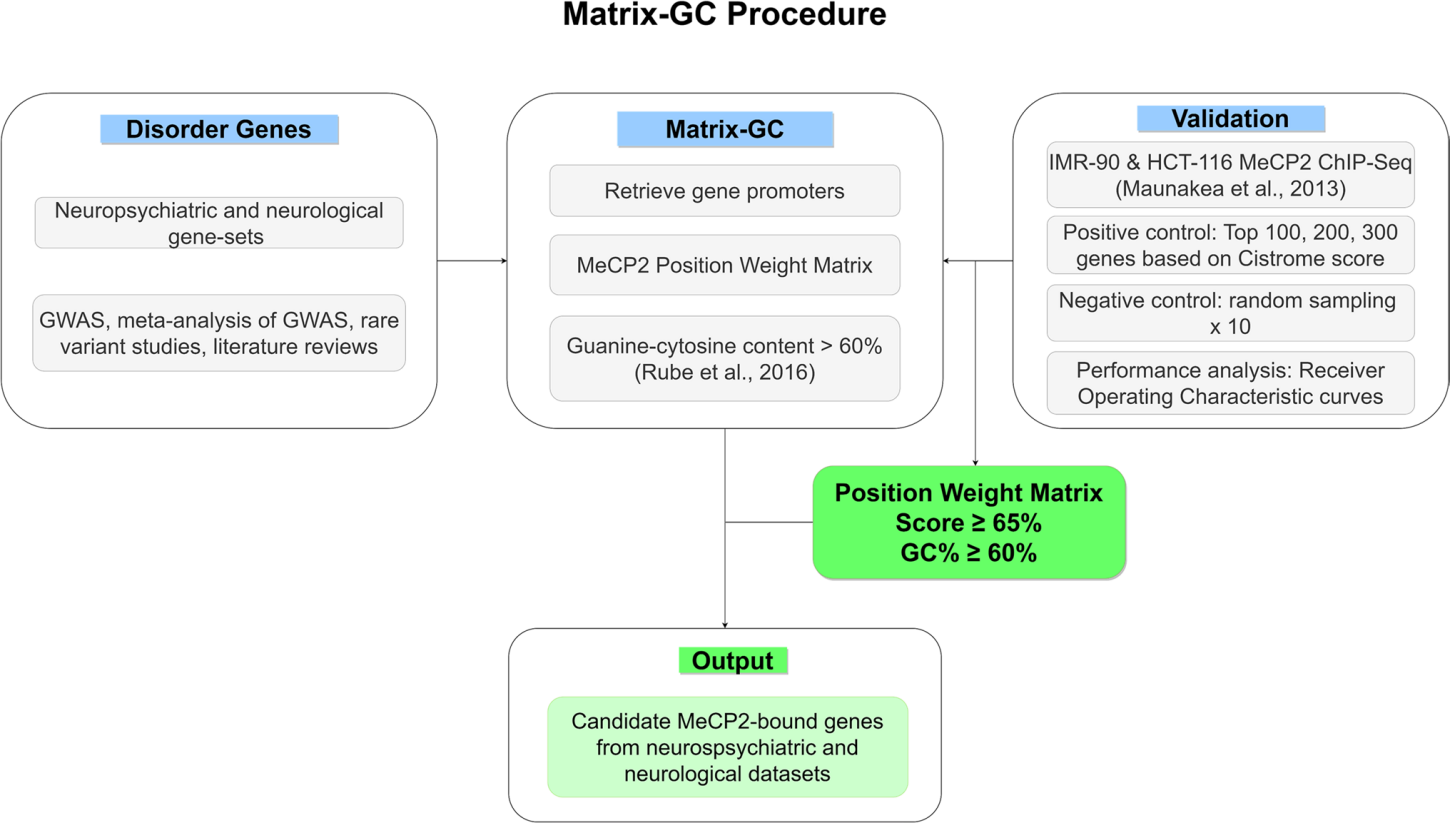
Overview of Matrix-GC procedure to detect MeCP2 binding sites in silico. The Matrix-GC procedure aims at identifying genes that are bound by MeCP2, through a combination of MeCP2 position weight matrix and DNA sequence GC%. We validate this procedure through positive and negative controls using ChIP-seq data (Maunakea et al., 2013) and evaluate its performance through Receiver Operating Characteristic curves. We apply Matrix-GC to the promoters of candidate genes across neurological and neuropsychiatric disorders to generate a list of putative genes bound by MeCP2 from each disorder.

We established a procedure to quantify MeCP2 binding in silico using the combination of a position weight matrix (PWM) and DNA sequence GC content (Fig. 1).

We established a position frequency matrix (PFM) for MeCP2 from the Cistrome database (http://cistrome.org)^23^. We used the Biostrings package in RStudio version 1.1.463, to convert the MeCP2 PFM into a PWM used to identify the MeCP2 binding motif along a sequence of DNA. We used the preferred sequences for MeCP2 binding through methyl-SELEX^23^ and validated with genes known to be bound by MeCP2, *Bdnf* and *Dlx6* in the promoter and core gene regions.

We retrieved MeCP2 target genes from ChIP-Seq data Cistrome Data Browser (http://cistrome.org/db/), and we used two sets of ChIP-Seq data from a study by Maunakea and colleagues (Cistrome ID 34,392 & 34,399)^24^. MeCP2′s target genes on Cistrome are already scored by the BETA package indicating the regulatory potential as a putative target^25^.

For positive controls, we generated sequence datasets for the top 100, 200 and 300 genes bound by MeCP2 from the IMR-90 and HCT-116 ChIP-Seq data, ranked by Cistrome BETA scoring. For negative controls, we randomly selected and size-matched genes from the same ChIP-Seq data with a score of 0. We define the promoter sequences as being 1000 bp upstream of the transcription start site and retrieved these promoters in RStudio from the UCSC Genome Browser (https://genome.ucsc.edu/) using the GRCh37/hg19 human reference genome. We tested each sequence for the presence of the MeCP2 PWM. For every PWM match, a score is given from 1–100%. This score represents how similar the motif of the PWM is on the selected sequences, compared to a random sequence.

Since Guanine-Cytosine nucleotide content (GC%) was previously established to be important in MeCP2 binding in vivo^26^, for every PWM match, we generated a sequence to include the 15 bp PWM match sequence and 100 bp flanking sequences, and we calculate the GC% for these 215 bp sequences.

### Receiver operating characteristics curve

In order to determine the ideal PWM threshold for MeCP2 motif binding, we graph a receiver operating characteristics (ROC) curve for all datasets. We set the minimum PWM score at 5% and stratified results based on PWM scores at increasing increments of 5%. We generated 10 random bootstrapped samples of 100, 200 and 300 negative control genes. Taking the average values, we plotted the ROC curve alongside the positive controls. Additionally, we evaluated ROC curves at various sequence GC%.

As additional validity controls, we consider the binding of the *CDKL4* gene as a negative control^27^ and *S100A9 as a* positive control^28,29^. Our Matrix-GC procedure captures the same findings in mouse *Cdkl4* and human *CDKL4* orthologue and *S100A9*.

### Dataset collection

For the analysis of MeCP2 interaction in different disorders, we used neuropsychiatric and neurological disorders data gathered from multiple studies (Supplementary Table S1). For the ASD- SFARI dataset^30^ Gene Scoring—which assesses the strength of evidence presented for candidate ASD genes—we considered categories S (syndromic), 1 (high confidence genes) and 2 (strong candidate genes).

For SCZ associated genes, we used genes identified by GWAS studies^22^. Rare variants are also implicated in SCZ aetiology, however at the moment, few candidate rare variants in SCZ have been confirmed with sequencing. Neurexin 1 is a well-known CNV in SCZ and is also largely associated with ASD^31^. To date, SETD1A is the only genome-wide significant rare variant discovered by whole exome sequencing^32^. The identification of rare variants in SCZ is controversial, so to avoid introducing false positive results in our study, we do not consider SCZ CNVs in our analysis.

To control for MeCP2 target genes involved in synaptic and immune function, we use genes as categorised by Lips and colleague^33^ and genes from the ImmPort data repository (https://www.immport.org/home), respectively.

### Enrichment and network analyses

We employed Gene Ontology using GORilla^34^, Reactome overrepresentation using ReactomePA R package^35^ and network analysis, to define functional aspects of the brain disorder gene datasets, and identify terms or pathways that are significantly enriched in these gene-sets. To validate our results, we use permutation analysis on control datasets randomly generated and size-matched, from hg19 human reference genome and exome subset. These datasets varied in size: 10, 20, 50, 100, 200, 500 and 750 genes. The terms and pathways significantly enriched from the analysis of the control datasets were excluded from the results of the genes associated with brain disorders in case of overlapping. For protein interaction analysis, we used Cytoscape and the stringApp plugin. We used randomly generated control datasets matching in gene numbers with the datasets from the brain disorders to identify the average degree of network connectivity related to the size of the datasets and to generate a range of values network connectivity associated to the size of the datasets. For each dataset size we run the Cytoscape analysis 20 times and we select the maximum and minimum degree of connectivity for each gene sets size across all the random analyses. This information was used to identify the brain disorders associated gene sets with a level of network connectivity different from what expected by chance. We considered protein hubs those with a degree of connection superior of at least 1 with respect to the average level of connectivity of the corresponding gene sets size. Only the hubs from the significant network are reported in this study.

### Tissue expression analysis

We look at the expression levels of each gene from our brain disorder gene-sets using NCBI.

Gene (https://www.ncbi.nlm.nih.gov/gene/). We select “HPA RNA-seq for normal tissues” for analysing protein-coding genes and “RNA sequencing of total RNA from 20 human tissues” for retrieving expression data of non-coding genes. Expression data is represented as reads per kilo base per million mapped reads.

In each tissue we considered a gene to be expressed if its expression level is greater than 0. This convention allows to obtain, for each disorder-tissue combination, a two-by-two contingency table of the number of genes that are (i) MeCP2-bound and expressed (MeCP2-bound genes are the genes selected by the MATRIX-GC procedure), (ii) MeCP2-bound and not expressed, (iii) not MeCP2-bound and expressed, and (iv) not MeCP2-bound and not expressed. From such a contingency table, Fisher’s exact test calculates from the hypergeometric distribution of the odds ratio the exact (i.e., finite sample rather than asymptotic) statistical significance of the hypothesis that the proportion of expressed genes in the MeCP2-bound group is the same as the proportion of expressed genes^36^ in the not MeCP2-bound group .

### Single nucleotide polymorphism in different brain disorders

To identify the presence of *MECP2* SNPs in the brain disorders considered, we downloaded human SNP data from NCBI dbSNP (https://www.ncbi.nlm.nih.gov/snp/). We compared *MECP2* SNPs to SNP from our brain disorder datasets of interest using data from NCBI ClinVar (https://www.ncbi.nlm.nih.gov/clinvar/). We also look at Matrix-GC-derived genes and investigated if SNPs were present (Supplementary Table S4). Sex information of patients with reported *MECP2* SNPs was derived from RettBASE: RettSyndrome.org Variation Database (mecp2.chw.edu.au).

### Functional validation using Transcriptomic data

To validate our results, we used transcriptomic analyses in *Mecp2*-null mice and RTT iPSCs. We used data from *Mecp2*- null mice compared to matched WT controls^28^ considering expression analysis in blood and cerebellum tissues, and data from iPSCs from a patient with Rett Syndrome^37^. Data was retrieved from the Gene Expression Omnibus under entries GSE129387 and GSE123753.

For the gene set identified by the Matrix-GC procedure, we calculated the percentage of significant DEGs (*p* ≤ 0.05) The DEGs were identified with EdgeR package (v3.14.0). Genes were not considered where all samples showed no counts.

We evaluated the statistical significance of these sets through a Monte Carlo method, by comparing their statistics to the percentage of DEGs (*p* ≤ 0.05) in 1000 randomly selected sets of genes with equal size. These Monte Carlo samples were selected from the set of *Mus musculus* orthologue genes for the animal studies, and from the brain tissue genes in the human studies.

## Results

### Establishing a high affinity MeCP2 binding procedure

**Figure 2:**
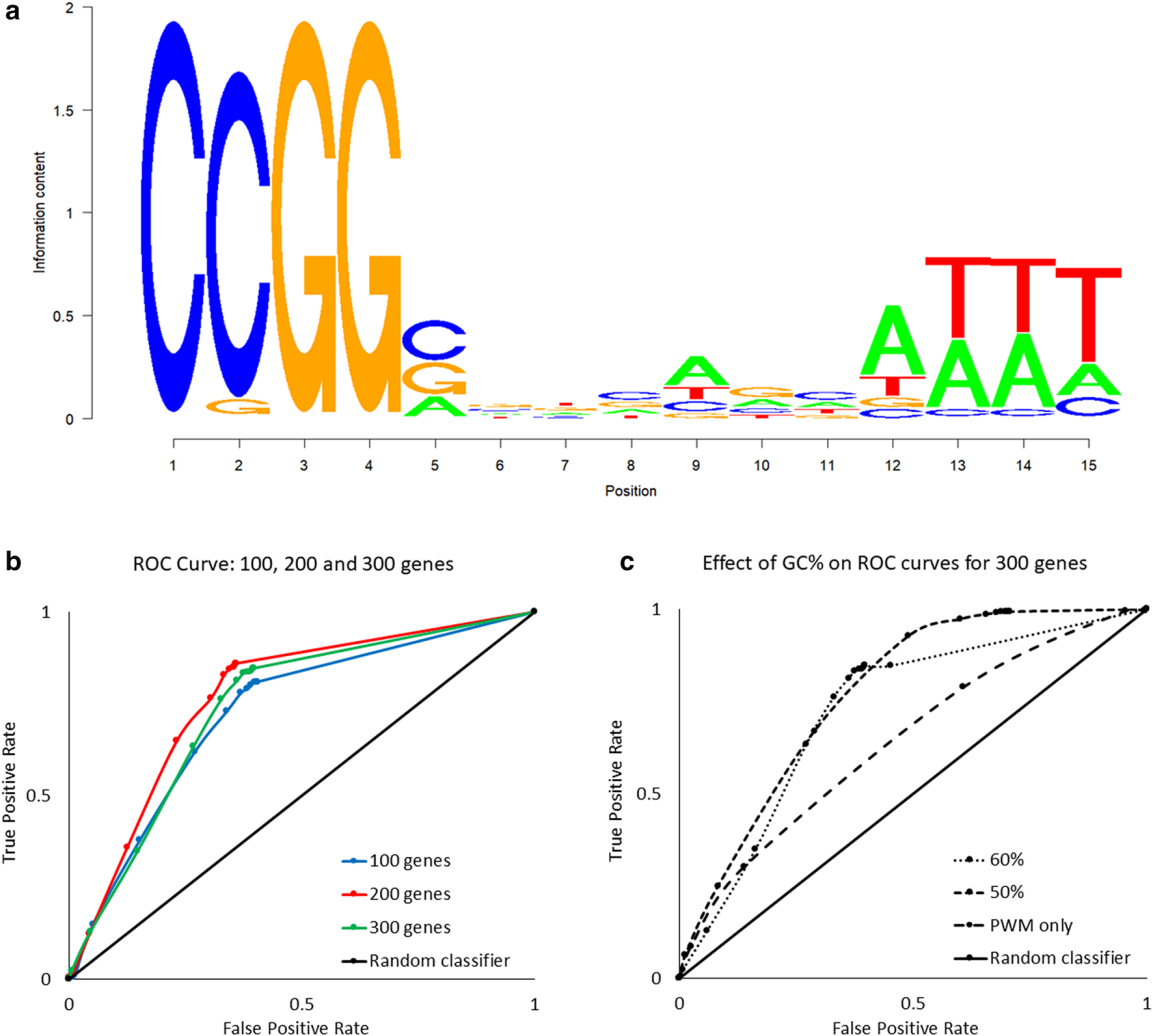
Construction of MeCP2 position weight matrix (PWM) threshold and GC%: Matrix-GC Procedure using MeCP2 ChIP-Seq data on IMR-90 cells. (**a**) Sequence logo for the conservation sequence for MeCP2. (**b**) ROC curves for 100, 200 and 300 genes establishing a preferential PWM score threshold. The area under the curve (AUC) is 0.725, 0.7685, 0.7419, for 100, 200 and 300 genes respectively. (**c**) ROC curves for 300 genes evaluating the effects of DNA sequence GC content percentage. The AUC values are 0.7301, 0.7692 and 0.6351 for GC content percentages of 50%, 60%, and PWM only, respectively. The random classifier is represented by x = y and has an AUC of 0.50.

To identify candidate target genes for MeCP2, we consider both nucleotide sequences, and the content of GC in promoters. Our procedure implements a PWM used to identify MeCP2 preferred sequences^23^ (Figs. 1, 2a), combined with GC content percentage of the promoter sequence to the optimal range of the selected variables.

To verify that our PWM + GC% filter is effective in identifying genes bound by MeCP2, we apply the model to the top scored genes in the MeCP2 ChIP-Seq IMR-90 data^24^. We generate ROC curves from datasets of 100, 200 and 300 genes to determine if there is a preferential threshold at different ranking levels (Fig. 2b,c). We identify the ideal threshold score to be 65% for MeCP2 binding across gene-sets. Since MeCP2 has a higher binding potential for regions containing GC dinucleotide occurrence of ≥ 60%^26^, we tested whether the threshold score of 0.65 changed with different percentages of GC content. We combine the PWM filter with an additional GC content filter, varying the GC percentage threshold from 60%, to 50% and without filtering for GC content (PWM only). We determine that 60% GC content offers a reduction in false positive rate by nearly half (50%: 0.657 vs. 60%: 0362 false positive rate). We observe similar results when using HCT-116 ChIP-Seq data and we confirm that a GC content of 60% is appropriate and in line with Rube and colleagues’ report^26^. For further analyses we use a PWM score of 65% and GC content of 60% (Matrix-GC). To confirm the validity of our procedure, we also test MeCP2 binding for a negative control (CDKL4,^27^) and positive control gene ( S100A9^29^).

We then examine MeCP2 binding potential according to Matrix-GC on gene-sets associated with neuropsychiatric and neurological disorders (Supplementary Table S1). All neuropsychiatric datasets have at least 50% of genes putatively bound by MeCP2 through the Matrix-GC procedure. Neurological datasets show an overall lower average percentage of MeCP2-bound genes (55.95%) compared to neuropsychiatric disorders (67.58%). These results suggest a higher involvement of MeCP2 in neuropsychiatric pathologies, although they can be attributed to the lower number of genes present the neurological datasets. We also consider the genome and applied Matrix-GC to all genes in the GRCh37/hg19 human reference genome, and we report an average of 39.56% genes bound by MeCP2 in silico across the genome (Supplementary Figure S1). We also investigate binding to synaptic and immune genes using our procedure and find that MeCP2 binds to 73.51% of synaptic genes and 44.87% of immune genes.

### Tissue expression of brain disorder-associated genes before and after matrix-GC

**Figure 3:**
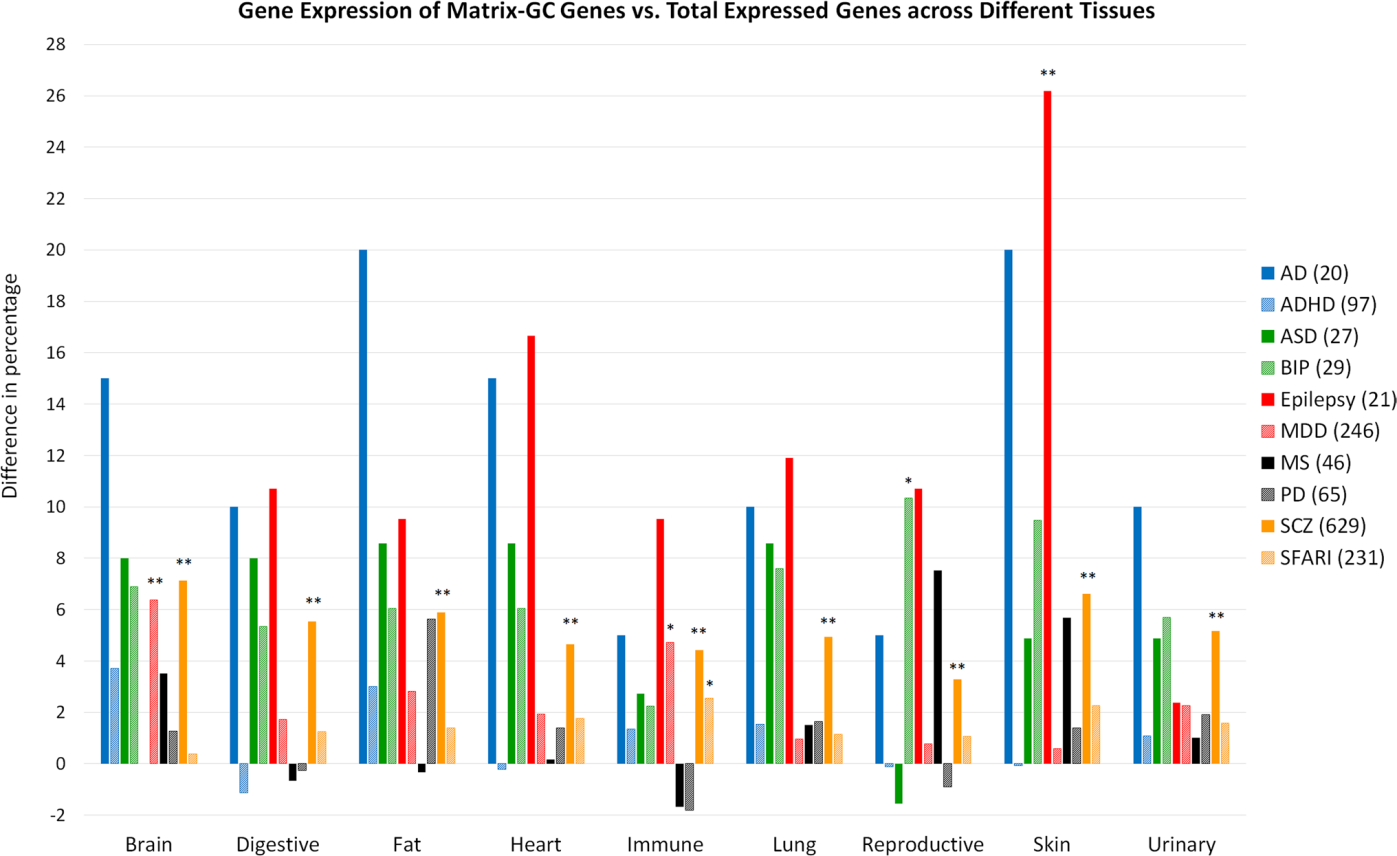
Genes associated to each disorder and tissue (horizontal axis) have a different RNA expression distribution (vertical, in percent) before and after Matrix-GC. Distribution of RNA expression, by tissue, for genes associated to brain disorders before and after Matrix-GC. The vertical axis reports the difference between the percentage of genes expressed in the different systems before and after Matrix-GC. Disorders included are Autism database (SFARI, Yellow), schizophrenia (SCZ, Orange), Parkinson’s disease (PD, Grey), multiple sclerosis (MS, Black), major depressive disorder (MDD, Pink), epilepsy (Red), autism (ASD, Dark Green), Alzheimer’s disease (AD, Blue), attention deficit hyperactivity disorder (ADHD, Light Blue) and bipolar disorder (BIP, Light Green). For immune, digestive, urinary and reproductive systems we considered data from different organs. Immune tissues include data from: lymph node, bone marrow, spleen, adrenal, thyroid and appendix data. Digestive includes data from: colon, duodenum, oesophagus, gall bladder, pancreas, liver, small intestine, salivary gland and stomach data. Reproductive tissues include data from: ovary, testis, endometrium, prostate and placenta. Urinary System urinary bladder and kidney data. Numbers in parentheses represent the total number of genes for which expression data was retrieved. The Fisher’s exact test was used for statistical calculations. *represents *p* value ≤ 0.05 and ** represents *p* value ≤ 0.01.

Using the NCBI Gene database, we investigate the expression of brain disorder-associated genes in different tissues before and after the Matrix-GC procedure (Fig. 3, Supplementary Table S2). There is a statistically significant difference in expression of brain disorders-associated genes in skin (epilepsy, *p* = 0.009), reproductive (BIP, *p* = 0.02), the brain (MDD, *p* = 0.0006), and immune (MDD, *p* = 0.01; SFARI, *p* = 0.02) tissues. SCZ shows a significant difference in genes bound by Matrix-GC and genes not bound by Matrix-GC in all tissue (Supplementary Table SS3).

ADHD genes show the largest increases in percentage after MatrixGC in brain (15%), fat (20%), and urinary tissues (10%). Expression in immune tissue for epilepsy genes increases by 10% after applying Matrix-GC. We also note differences after Matrix-GC, in lung (12%), heart (17%), and skin (26%), tissues for epilepsy-related genes.

We also consider distribution and expression of non-coding RNAs (ncRNAs) in our gene-sets (Supplementary Table S2). *PVT1* is the only ncRNA bound by MeCP2 in silico in MS. 5 ncRNAs are found in the ADHD dataset: T*MEM161B-AS1, LINC01572, LINC00461, KDM4A-AS1, LINC02060*, of which only the first 3 are positive by our Matrix-GC procedure. 28 out of 64 ncRNAs in the SCZ dataset are identified by Matrix-GC. There is no statistically significant difference between the number of genes expressed before or after Matrix-GC. However, we do report that there is an increase in percentage of expressed genes after Matrix-GC in the cerebellum (24.11%) and whole brain (22.32%). The role of these ncRNAs is unknown apart from association to disorders in GWAS studies. One limit of this analysis is the poor availability of data and the unknown developmental stage at the time of tissue collection for the analysis of the expression levels^38^.

### ***MECP2*** mutations are present in brain disorders

Using NCBI dbSNP, we find 13,100 SNPs in *MECP2* and few of them are present in four of our selected disorders: ADHD (2 SNPs), ASD (87 SNPs), epilepsy (25 SNPs) and SCZ (12 SNPs) (Supplementary Table S4). The presence of *MECP2* SNPs in SCZ and ASD is expected given *MECP2* is involved and well-studied in these disorders.

In epilepsy, the presence of *MECP2* SNPs in some patients is not surprising, considering the presence of epilepsy in 75% of the cases of RTT, although the effect of *MECP2* mutations in epilepsy are not well understood. The correlation between *MECP2* mutations and epileptic phenotype in RTT has proved a challenge to describe, due to the complex nature and presentation of the disorder^39^.

The possible involvement of MeCP2 in ADHD has not been properly established, despite the known relationship between ADHD and ASD (and by extension, MeCP2). However, the presence of *MECP2* SNPs in ADHD patients, suggests the involvement of MeCP2 in the pathology. This hypothesis is confirmed by immunofluorescence studies where *MECP2* expression is reduced in ADHD cerebral cortices^40^, and a more recent study looking at epigenetic biomarkers to predict ADHD diagnoses in children shows correlation between predictability and decreased *MECP2* mRNA levels^41^.

### Protein–protein interaction network analysis through cytoscape

To identify protein–protein interactions and central proteins or nodes that are highly connected in each disorder, we use Cytoscape and the stringApp, and input the MeCP2-bound genes filtered with the Matrix-GC procedure (Table 1, Supplementary Table S5).

**Table 1:** Top 30 hub proteins, ranked by node degree, from protein–protein interaction analysis.

Protein	Degree	Name	Condition
EP300	45	Protein propionyltransferase p300	SFARI
CHD8	44	Chromodomain helicase DNA binding protein 8	SFARI
MECP2	31	Methyl CpG binding protein 2	SFARI
PTEN	30	Mutated in multiple advanced cancers 1	SFARI
KMT2C	28	Myeloid/lymphoid or mixed-lineage leukemia protein 3	SFARI
SIN3A	27	SIN3 transcription regulator family member A	SFARI
KDM6A	26	Ubiquitously-transcribed X chromosome tetratricopeptide repeat protein	SFARI
GRIN2B	26	Glutamate receptor, ionotropic, N-methyl D-aspartate 2B	SFARI
UBE3A	23	Human papillomavirus E6-associated protein	SFARI
SETD1B	23	Histone-lysine N-methyltransferase SETD1B	SFARI
NF1	23	Neurofibromatosis-related protein NF-1	SFARI
SLC6A3	22	Solute carrier family 6 (neurotransmitter transporter), member 3	ADHD
YY1	22	Transcriptional repressor protein YY1	SFARI
SMARCA2	22	SWI/SNF related, matrix associated, actin dependent regulator of chromatin, subfamily a, member 2	SFARI
HDAC4	22	Histone deacetylase 4	SFARI
GRM5	21	Glutamate receptor, metabotropic 5	ADHD
KAT2B	21	Histone acetyltransferase KAT2B	SFARI
DNMT3A	21	DNA (cytosine-5-)-methyltransferase 3 alpha	SFARI
MTOR	20	FK506-binding protein 12-rapamycin complex-associated protein 1	SFARI
KMT2A	20	Myeloid/lymphoid or mixed-lineage leukemia protein 1	SFARI
FMR1	20	Fragile X mental retardation protein 1	SFARI
GRIN2B	19	Glutamate receptor, ionotropic, N-methyl D-aspartate 2B	ADHD
SHANK2	19	SH3 and multiple ankyrin repeat domains protein 2	SFARI
CNTNAP2	19	Contactin associated protein-like 2	SFARI
ASH1L	19	Ash1 (absent, small, or homeotic)-like (Drosophila)	SFARI
SYNGAP1	18	Ras/Rap GTPase-activating protein SynGAP	SFARI
SMARCC2	18	SWI/SNF related, matrix associated, actin dependent regulator of chromatin, subfamily c, member 2	SFARI
PPP2CA	18	Serine/threonine-protein phosphatase 2A catalytic subunit alpha isoform	SFARI
POGZ	18	Pogo transposable element with ZNF domain	SFARI
PAX6	18	Aniridia type II protein;	SFARI

Degrees obtained from Cytoscape and stringApp plugin. The Hub proteins shown are from ADHD and Autism SFARI datasets. (Such datasets have a statistically significant network connectivity compared to controls.) A node is designated as hub if its degree is greater than one plus the average degree of the corresponding control group. The entire list of hub proteins is in the Supplementary Information Files.

We generated control gene sets to identify the average degree of network connectivity depending on the number of genes, and we use this information to identify the Matrix-GC gene sets with a significant degree of connectivity (Supplementary Fig. S2, Tables S6). We show that AD, ADHD, MS, and SFARI datasets before and after applying Matrix-GC show statistically significant connected networks. From our protein–protein interaction (PPI) network, we identify hub proteins from the Matrix-GC datasets with a significant connectivity. After Matrix-GC, ADHD hub proteins are associated with neurotransmission processes and different neurotransmitter systems such as DRD1, DRD4, DRD5 dopamine receptors, and GRM5, GRIN2B glutamate receptors. MS-designated hub proteins are involved in eliciting an inflammatory response such as TYK2, STAT3, CD40. Hub proteins in AD are generally associated with cell communication while in SFARI, the most connected proteins are involved in DNA processes, namely transcription. Notably, EP300 is a hub protein with the highest degree out of all disorders. EP300 is a histone acetylase regulated indirectly by MeCP2 likely via MEF2C^42^.

Overall, the results of the PPI analysis highlight proteins involved in inflammatory responses, transcription regulation and neurotransmission.

### Enrichment analysis reveals unexpected MeCP2 influence in neuropsychiatric and neurological disorders

**Figure 4:**
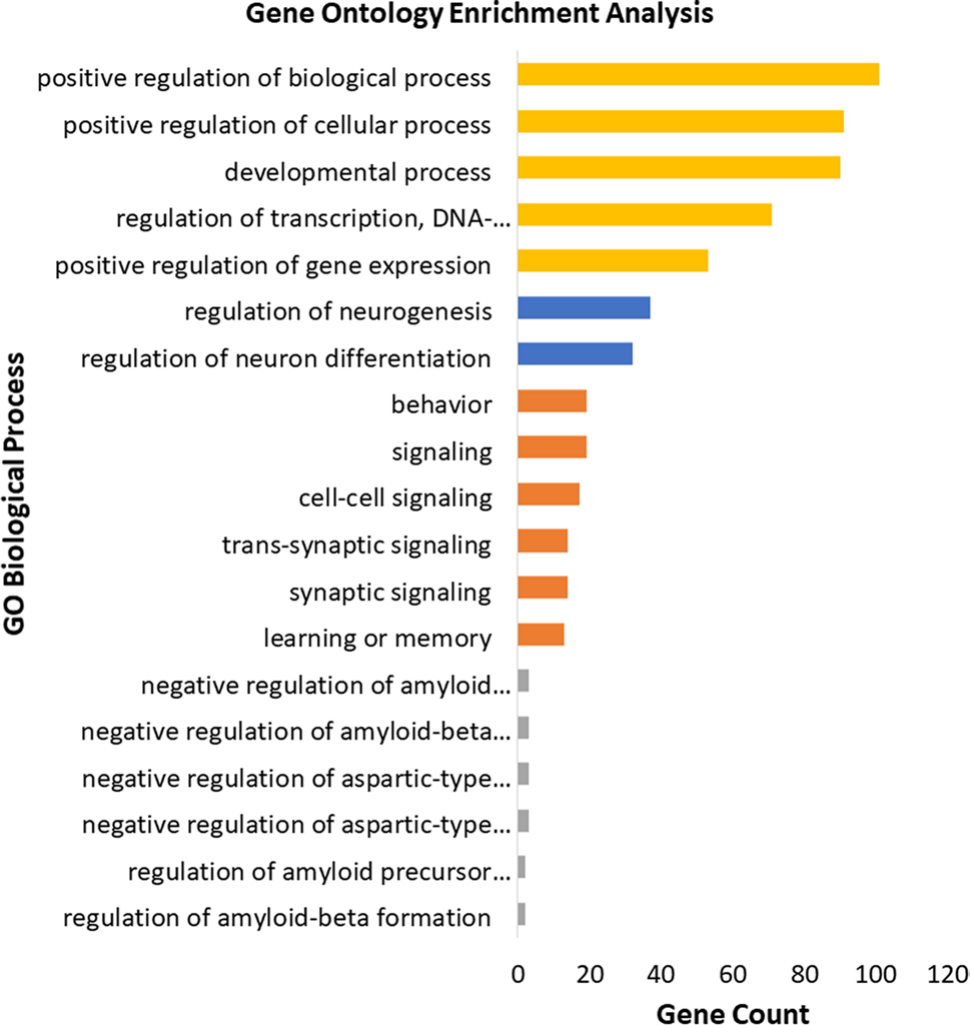
Gene Ontology enrichment analysis of neuropsychiatric and neurological disorders using GOrilla Enrichment. Bar plot of the 5 most significant results from Gene Ontology enrichment analysis of each neuropsychiatric and neurological datasets after the Matrix-GC. The following disorders are represented: attention deficit hyperactivity disorder (ADHD, Orange), schizophrenia (SCZ, Blue), Alzheimer’s disease (AD, Grey), Autism database (ASD-SFARI, Yellow). Full statistical results from GOrilla Enrichment can be found in the Supplementary Information files. Control permutation analysis was carried out on randomly generated and size-matched, from hg19 human reference genome and exome subset*.*

We then carry out GO and pathway enrichment analysis before and after the application of Matrix-GC, and in three of the selected datasets (ADHD, AD and ASD-SFARI), we find significant GO Biological Process terms before and after the binding procedure. To control for false positives, we carried out permutation analysis with control datasets randomly selected from the genome and exome. SCZ GO terms are significant after Matrix-GC only while for epilepsy, MDD, MS, and PD-related genes there are significant terms prior to Matrix-GC only (Fig. 4, Supplementary Tables S7-S19). Over-representation analysis shows that terms related to neuronal growth, differentiation and nervous system development are significantly enriched in both ADHD and SCZ datasets. Additionally, ADHD-related genes show significant enrichment in behaviour and learning, cell–cell communication, and catecholamine neurotransmission and metabolism. AD-related genes detected by the Matrix-GC procedure show significantly enriched terms related to amyloid protein regulation, metabolism, protein filaments and endocytosis. The ASD-SFARI dataset has the highest number of enriched terms before and after the Matrix-GC procedure, and the most significant terms relate to nucleic acid processes. However, enrichment and network analysis based on common variants does not identify terms or pathways in the ASD-GWAS gene-set. It is possible that MeCP2 plays a role in ASD by coordinating functional connectivity and controlling neurotransmitter balance and cell growth as seen in other neuropsychiatric disorders^10^.

**Figure 5:**
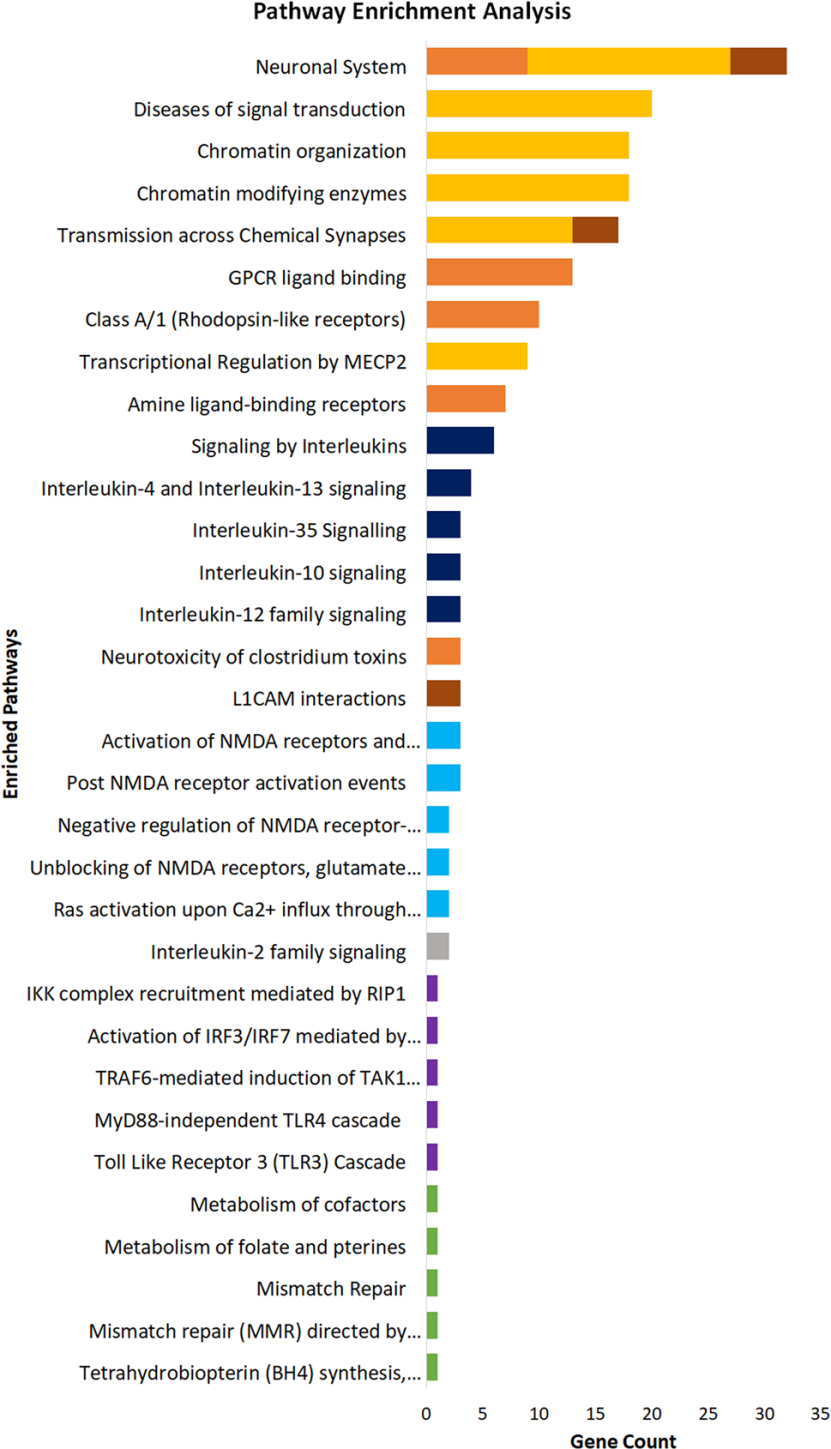
Pathway enrichment analysis of neuropsychiatric and neurological disorders using ReactomePA. Bar plot of the 5 most significant results from pathway enrichment analysis of each neuropsychiatric and neurological datasets after Matrix-GC. The following disorders are represented: attention deficit hyperactivity disorder (ADHD, Orange), bipolar disorder (BIP, Brown), Alzheimer’s disease (AD, Grey), multiple sclerosis (MS, Dark Blue), Parkinson’s disease (PD, Light Blue) Huntington’s disease (HD, Green) and amyotrophic lateral sclerosis (ALS, Purple), autism database (ASD-SFARI, Yellow). Full statistical results from ReactomePA can be found in the Supplementary Information files. Control permutation analysis was carried out on randomly generated and size-matched, from hg19 human reference genome and exome subset.

For the analysis of pathways, we use Reactome (Fig. 5, Supplementary Tables S7-S19). Only MDD, anorexia and epilepsy gene-sets display enriched pathways solely before our Matrix-GC procedure, while SCZ and ASD-GWAS gene-sets have no enriched pathways either before or after the Matrix-GC procedure. Conversely, PD pathways are significant after Matrix-GC only, and ASD-SFARI, AD, ADHD, ALS, BIP, HTT and MS have enriched pathways both before and after the procedure.

The most significantly enriched pathway across all investigated disorders is amine ligand-binding receptors in ADHD (adjusted *p* value = 8.12 × 10^–8^). Glutamate and CREB related pathways are also enriched in ADHD-related genes, while the ASD-SFARI dataset has the highest number of overrepresented pathways associated with chromatin organisation, growth and neurotransmitter processes. AD and MS datasets are significantly enriched for interleukin signalling pathways before and after Matrix-GC procedure. ALS-related genes are enriched for Toll-like receptor processes before and after Matrix-GC, while PD-related genes are significantly associated to NMDA-related pathways and BIP genes are enriched for neuronal development and functional pathways.

For Gene Ontology and pathway analysis, we use Monte Carlo permutation tests on randomly-generated control datasets to identify potential false positives. We report for both genome and exome datasets, that no pathways or terms are significant after 1000 trials, confirming our results.

Overall, we find the ADHD, AD and ASD-SFARI derived genes to be highly enriched for multiple GO terms and Reactome pathways, suggesting that several mechanisms controlled by MeCP2 are relevant in these disorders.

### Functional validation of MeCP2 modulation of candidate genes

To prove that the candidate genes identified through our procedure are indeed target of MeCP2, we evaluate their expression levels in a mouse mutant for *Mecp2* (*Mecp2*^tm1.1Bird^ data from Cerebellum and Blood (GSE129387), and in cells derived from a patient with RTT (GSE123753). The male mice of this strain are *Mecp2* knockouts, hence the effects of MeCP2 binding on the Matrix-GC genes can be more clearly evaluated. We reasoned that the expression of the genes directly affected by MeCP2 should be altered in the mutant mice compared to the matched control and we evaluated the expression of the candidate genes in blood and brain. We considered the expression levels in mutant mice and matched controls, and in particular, we looked at the percentage of significant DEGs (*p* value ≤ 0.05). We considered all the Matrix-GC genes across the disorder datasets. Of the total 1018 genes bound by our procedure, 380 genes are from cerebellum, 446 genes are from blood and 301 from the cortex when cross-referenced with GTEx portal single-tissue eQTL data.

**Figure 6:**
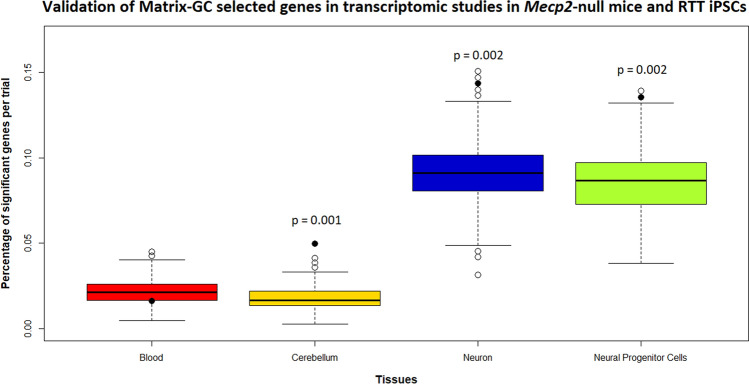
Validation of Matrix-GC selected genes in transcriptomic studies in Mecp2-null mice and RTT iPSCs. Distribution of significant genes (vertical axis, in percent) against experimental groups (horizontal): blood in *Mecp2*^tm1.1Bird^ mice (Red), cerebellum in *Mecp2*^tm1.1Bird^ mice (Yellow), iPSCs-derived neurons from RTT patient (Blue, *MECP2* Del ex 3–4 mutation), iPSCs-derived Neural Progenitor Cells from RTT patient (Green, *MECP2* Del ex 3–4 mutation). Monte Carlo permutation analysis yields the percentage of significant genes (*p* value ≤ 0.5) on 1000 randomly generated and sized-matched control gene-sets. The line within the box represents the median of the distribution. The top and bottom edges of the box represent the 3rd quartile (Q3) and 1st quartile (Q1) respectively. The upper and bottom whiskers represent Q3 + 1.5 times interquartile range, and Q1 – 1.5 times the interquartile range. Empty circles represent the outlier observations in each group, solid circles the percentage of Matrix-GC filtered genes in each group. *p* values are reported above groups where Matrix-GC filtered genes are significant among controls.

The percentage of significant genes expressed in the mouse brain and blood was 0.049% and 0.017% respectively. Monte Carlo analysis did not show any significant results in the blood, but rather in the cerebellum (*p* value < 0.01) suggesting that the role of MeCP2 is more important in the brain than in the blood. For the validation in RTT iPSCs, we considered the study in a RTT patient with a mutation comparable to the one in the mouse and used a transcriptomic study in iPSCs cells from a patient with a deletion in exons 3 and 4 in the *MECP2* gene^37^. The percentage of genes expressed with a *p* value ≤ 0.05 in neural progenitor cells and neurons was 14.69% and 13.54% respectively. The statistical analysis revealed significant results both in neural progenitor cells and in differentiated neurons (Fig. 6).

For each study we controlled for the specificity of our results using controls from corresponding tissues and species: using permutation analysis we generated 1000 random control datasets of the same size of the total candidate list, and we considered the distribution of the percentage of genes whose expression was significantly different between mutants and controls (*p* value ≤ 0.05). By looking at the distribution of the data in controls datasets, we confirmed that the expression change of our MeCP2-candidate target genes was in the 1% of the distribution, supporting the validity of our method to select genes directly modulated by MeCP2 (Fig. 6).

## Discussion

Our work proposes that MeCP2 binds many genes associated with brain disorders and is involved in overlapping molecular mechanisms between conditions. These findings invite us to revisit the molecular aetiology of brain disorders and suggest that therapies that affect MeCP2 function may be effective not only for Rett Syndrome, but also for other pathologies.

*MECP2* mutations have been associated to several pathologies, especially neuropsychiatric disorders, but to date there is no direct proof of MeCP2 modulation of genes associated to brain disorders. Our results suggest that MeCP2 takes part in mechanisms associated with several brain disorders, not only through its action on synapses,^43^ but also by binding genes mediating other functions, including inflammation. The value of our Matrix-GC procedure for identifying MeCP2 target genes is reinforced by the functional validation in transcriptomics studies in *Mecp2* mutant mice and on iPSCs derived from a patient with RTT.

Among the disorders considered, we find that SNPs in *MECP2* are present in SCZ, epilepsy, ASD-GWAS and ADHD, although the downstream enrichment analysis indicates that MeCP2 is also involved mechanisms in other disorders. Several correlations between *MECP2* expression and brain disorder mechanisms have been reported in the literature. This can occur directly through *MECP2* mutations^44–46^ or indirectly via MeCP2 regulating BDNF^2,3^, and ncRNA action^47–49^.

Although the majority of results are associated to neuropsychiatric disorders, our enrichment analysis suggests an interaction between MeCP2 and neurological conditions such as Alzheimer disease, multiple sclerosis and epilepsy. Our enrichment analysis suggests that such interaction is mediated by neuroinflammation. Inflammation is already implicated in AD and its progression^50^ and is also critical in MS pathology. Similar to MS, RTT displays features that are hallmarks of autoimmune disorders suggesting potential common therapeutics^51–53^. We observe an effect of MeCP2 on immune-related genes, and in particular to S100A9, a gene already identified in transcriptomic studies on blood and brain of *Mecp2*-null mice^29^. The levels of S100A8 and S100A9 proteins are related to inflammation, and are elevated in MS and AD.

Overall our analysis proposes three main mechanisms mediated by MeCP2 in different disorders: neuronal transmission, immune-related pathways and processes for growth and development.

The Reactome and GO outputs include dopaminergic and glutamatergic related terms and pathways in ADHD, SFARI, and PD gene-sets, and this result is reinforced by hub proteins in the PPI network analysis related to DA and glutamate receptors in ADHD and SFARI. Dopaminergic dysregulation in RTT patients has been observed through reductions in DA or its metabolite, homovanillic acid^54,55^. This dysregulation leads to dyskinesia, hand stereotypies and rigidity: symptoms found also in RTT. Alteration in dopamine transmission is a feature of several neurological disorders, notably PD, but also in AD, ADHD, SCZ, MS and HTT^56–59^.

Increased levels of glutamate^60^ are observed in patients with RTT and animal models^60,61^ and, glutamatergic synapses are regulated by MeCP2^62^. NMDA receptor-related Reactome pathways are enriched in ADHD and PD gene-sets. Additionally, several MeCP2-target hub proteins in PPI analysis are present in SCZ-associated dataset, and relate to DA and glutamate receptors^63^. These results suggest that MeCP2′s influence in dopaminergic and glutamatergic systems has functional and behavioural consequences in several brain disorders.

MeCP2 action on several systems is suggested by our tissue expression analysis, which reports that genes modulated by MeCP2 are expressed not only in the nervous system, but in other systems such as the immune system, and AD, ALS and MS gene-sets are enriched for interleukin and Toll-like receptor signalling pathways. Tissue gene expression analysis of AD genes show an increase in immune tissues after Matrix-GC, confirming pathway enrichment results, and MeCP2 is reported to alter T-lymphocyte gene expression profile^64^. Altered immunity has also been reported in neuropsychiatric disorders such as SCZ, depression and ASD^65,66^. Interestingly, we observe one immune GO term (GO:0,002,292, T-cell differentiation involved in immune response) in the ASD-SFARI dataset.

Growth and developmental processes appear across different datasets in enrichment analysis with terms and pathways related to cell cycle and proliferation. This association is not surprising, given that MeCP2 is an epigenetic modifier in cancer^67^. In our analysis, two antisense RNAs *EP300-AS1* and *MEF2C-AS1* in SCZ are correlated to MeCP2, and *MEF2C-AS1* has the highest expression in the cerebellum. EP300 is also a hub protein detected in our PPI network analysis: a histone acetylase regulated indirectly by MeCP2 likely via MEF2C, which is a transcription factor that binds to the MeCP2 promoter and controls MeCP2 expression^42^. MEF2C mutations affect MeCP2 function and this has been observed in epilepsy and ADHD’s studies^68^. Taken together, these results suggest that MeCP2 exerts influence in early development in SCZ and ASD. Similarly, the ADHD dataset is enriched for pathways related to neurotrophic factor signalling which mediates neuronal proliferation and maturation^69^.

Overall, our results propose a direct and indirect contribution of MeCP2 to mechanisms linked to several brain disorders. Additional experimental evidence would reinforce this hypothesis and may suggest common therapeutic targets across different conditions.

## Supplementary information

Supplementary Tables.

## Data Availability

All the data supporting the results of this study are available within this article and in the Supplementary Information files. The detailed procedure and R scripts used in the analysis supporting the findings of this study are available from the corresponding author upon reasonable request.

## References

[1] Chen WG (2003). Derepression of BDNF transcription involves calcium-dependent phosphorylation of MeCP2. Science (80-).

[2] Srivastav S, Walitza S, Grünblatt E (2018). Emerging role of miRNA in attention deficit hyperactivity disorder: a systematic review. ADHD Atten. Deficit Hyperact. Disord..

[3] Autry AE, Monteggia LM (2012). Brain-derived neurotrophic factor and neuropsychiatric disorders. Pharmacol. Rev..

[4] Makedonski K, Abuhatzira L, Kaufman Y, Razin A, Shemer R (2005). MeCP2 deficiency in Rett syndrome causes epigenetic aberrations at the PWS/AS imprinting center that affects UBE3A expression. Hum. Mol. Genet..

[5] Coffee B, Zhang F, Ceman S, Warren ST, Reines D (2002). Histone modifications depict an aberrantly heterochromatinized FMR1 gene in fragile X syndrome. Am. J. Hum. Genet..

[6] Khwaja OS, Sahin M (2011). Translational research: Rett syndrome and tuberous sclerosis complex. Curr. Opin. Pediatr..

[7] Wang Y (2017). Repression of TSC1/TSC2 mediated by MeCP2 regulates human embryo lung fibroblast cell differentiation and proliferation. Int. J. Biol. Macromol..

[8] Li H, Yamagata T, Mori M, Yasuhara A, Momoi MY (2005). Mutation analysis of methyl-CpG binding protein family genes in autistic patients. Brain Dev..

[9] Suter B, Treadwell-Deering D, Zoghbi HY, Glaze DG, Neul JL (2014). Brief report: MECP2 mutations in people without Rett syndrome. J. Autism Dev. Disord..

[10] Ausió J, de Paz AM, Esteller M (2014). MeCP2: the long trip from a chromatin protein to neurological disorders. Trends Mol. Med..

[11] Grove J (2019). Identification of common genetic risk variants for autism spectrum disorder. Nat. Genet..

[12] Hayman V, Fernandez TV (2018). Genetic insights into ADHD biology. Front. Psychiatry.

[13] Howard DM (2019). Genome-wide meta-analysis of depression identifies 102 independent variants and highlights the importance of the prefrontal brain regions. Nat. Neurosci..

[14] Stahl EA (2019). Genome-wide association study identifies 30 loci associated with bipolar disorder. Nat. Genet..

[15] Duncan L (2017). Significant locus and metabolic genetic correlations revealed in genome-wide association study of anorexia nervosa. Am. J. Psychiatry.

[16] Abou-Khalil B, Auce P, Avbersek A, Bahlo M, Balding DJ (2018). Genome-wide mega-analysis identifies 16 loci and highlights diverse biological mechanisms in the common epilepsies. Nat. Commun..

[17] Lambert JC (2013). Meta-analysis of 74,046 individuals identifies 11 new susceptibility loci for Alzheimer’s disease. Nat. Genet..

[18] Chang D (2017). A meta-analysis of genome-wide association studies identifies 17 new Parkinson’s disease risk loci. Nat. Genet..

[19] Moss DJH (2017). Identification of genetic variants associated with Huntington’s disease progression: a genome-wide association study. Lancet Neurol..

[20] van Rheenen W (2016). Genome-wide association analyses identify new risk variants and the genetic architecture of amyotrophic lateral sclerosis. Nat. Genet..

[21] Bashinskaya VV, Kulakova OG, Boyko AN, Favorov AV, Favorova OO (2015). A review of genome-wide association studies for multiple sclerosis: classical and hypothesis-driven approaches. Hum. Genet..

[22] Pardiñas AF (2018). Common schizophrenia alleles are enriched in mutation-intolerant genes and in regions under strong background selection. Nat. Genet..

[23] Klose RJ (2005). DNA binding selectivity of MeCP2 due to a requirement for A/T sequences adjacent to methyl-CpG. Mol. Cell.

[24] Maunakea AK, Chepelev I, Cui K, Zhao K (2013). Intragenic DNA methylation modulates alternative splicing by recruiting MeCP2 to promote exon recognition. Cell Res..

[25] Wang S (2013). Target analysis by integration of transcriptome and ChIP-seq data with BETA. Nat. Protoc..

[26] Rube HT (2016). Sequence features accurately predict genome-wide MeCP2 binding in vivo. Nat. Commun..

[27] Chahrour M (2008). MeCP2, a key contributor to neurological disease, activates and represses transcription. Science (80-).

[28] Sanfeliu A, Hokamp K, Gill M, Tropea D (2019). Transcriptomic analysis of Mecp2 mutant mice reveals differentially expressed genes and altered mechanisms in both blood and brain. Front. Psychiatry.

[29] Urdinguio RG (2008). Mecp2-null mice provide new neuronal targets for Rett syndrome. PLoS ONE.

[30] Abrahams BS (2013). SFARI Gene 2.0: a community-driven knowledgebase for the autism spectrum disorders (ASDs). Mol. Autism.

[31] Kim HG (2008). Disruption of neurexin 1 associated with autism spectrum disorder. Am. J. Hum. Genet..

[32] Singh T (2016). Rare loss-of-function variants in SETD1A are associated with schizophrenia and developmental disorders. Nat. Neurosci..

[33] Lips ES (2012). Functional gene group analysis identifies synaptic gene groups as risk factor for schizophrenia. Mol. Psychiatry.

[34] Eden E, Navon R, Steinfeld I, Lipson D, Yakhini Z (2009). GOrilla: A tool for discovery and visualization of enriched GO terms in ranked gene lists. BMC Bioinform..

[35] Yu G, He QY (2016). ReactomePA: An R/Bioconductor package for reactome pathway analysis and visualization. Mol. Biosyst..

[36] Fisher RA (1922). On the interpretation of χ 2 from contingency tables, and the calculation of P. J. R. Stat. Soc..

[37] Rodrigues DC (2020). Shifts in ribosome engagement impact key gene sets in neurodevelopment and ubiquitination in Rett syndrome. Cell Rep..

[38] Fagerberg L (2014). Analysis of the human tissue-specific expression by genome-wide integration of transcriptomics and antibody-based proteomics. Mol. Cell. Proteomics.

[39] Operto FF, Mazza R, Pastorino GMG, Verrotti A, Coppola G (2019). Epilepsy and genetic in Rett syndrome: a review. Brain Behav..

[40] Nagarajan RP, Hogart AR, Gwye Y, Martin MR, LaSalle JM (2006). Reduced MeCP2 expression is frequent in autism frontal cortex and correlates with aberrant MECP2 promoter methylation. Epigenetics.

[41] Xu Y (2015). Multiple epigenetic factors predict the attention deficit/hyperactivity disorder among the Chinese Han children. J. Psychiatr. Res..

[42] Zweier M (2010). Mutations in MEF2C from the 5q14.3q15 microdeletion syndrome region are a frequent cause of severe mental retardation and diminish MECP2 and CDKL5 expression. Hum. Mutat..

[43] Na ES, Nelson ED, Kavalali ET, Monteggia LM (2013). The impact of MeCP2 loss- or gain-of-function on synaptic plasticity. Neuropsychopharmacology.

[44] Liu Z (2016). Autism-like behaviours and germline transmission in transgenic monkeys overexpressing MeCP2. Nature.

[45] Peters SU (2013). The behavioral phenotype in MECP2 duplication syndrome: a comparison with idiopathic autism. Autism Res..

[46] Wong DF (2018). Are dopamine receptor and transporter changes in Rett syndrome reflected in Mecp2-deficient mice?. Exp. Neurol..

[47] Soreq H, Wolf Y (2011). NeurimmiRs: MicroRNAs in the neuroimmune interface. Trends Mol. Med..

[48] Visvanathan J, Lee S, Lee B, Lee JW, Lee SK (2007). The microRNA miR-124 antagonizes the anti-neural REST/SCP1 pathway during embryonic CNS development. Genes Dev..

[49] Yin J (2014). MiR-137: a new player in schizophrenia. Int. J. Mol. Sci..

[50] Kinney JW (2018). Inflammation as a central mechanism in Alzheimer’s disease. Alzheimer’s Dement. Transl. Res. Clin. Interv..

[51] De Felice C (2016). Rett syndrome: An autoimmune disease?. Autoimmun. Rev..

[52] Baker D (2001). Endocannabinoids control spasticity in a multiple sclerosis model. FASEB J..

[53] Vigli D (2018). Chronic treatment with the phytocannabinoid Cannabidivarin (CBDV) rescues behavioural alterations and brain atrophy in a mouse model of Rett syndrome. Neuropharmacology.

[54] Zoghbi HY (1989). Cerebrospinal fluid biogenic amines and biopterin in Rett syndrome. Ann. Neurol..

[55] Lekman A (1989). Rett syndrome: Biogenic amines and metabolites in postmortem brain. Pediatr. Neurol..

[56] Wolfe N (1990). Neuropsychological profile linked to low dopamine: in Alzheimer’s disease, major depression, and Parkinson’s disease. J. Neurol. Neurosurg. Psychiatry.

[57] Dobryakova E, Genova HM, DeLuca J, Wylie GR (2015). The dopamine imbalance hypothesis of fatigue in multiple sclerosis and other neurological disorders. Front. Neurol..

[58] Volkow ND (2009). Evaluating dopamine reward pathway in ADHD: clinical implications. J. Am. Med. Assoc..

[59] Money KM, Stanwood GD (2013). Developmental origins of brain disorders: roles for dopamine. Front. Cell. Neurosci..

[60] Lappalainen R, Riikonen RS (1996). High levels of cerebrospinal fluid glutamate in Rett syndrome. Pediatr. Neurol..

[61] Maezawa I, Jin L-WW (2010). Rett syndrome microglia damage dendrites and synapses by the elevated release of glutamate. J. Neurosci..

[62] Meng X (2016). Manipulations of MeCP2 in glutamatergic neurons highlight their contributions to Rett and other neurological disorders. Elife.

[63] Howes O, McCutcheon R, Stone J (2015). Glutamate and dopamine in schizophrenia: an update for the 21st century. J. Psychopharmacol..

[64] Delgado IJ, Kim DS, Thatcher KN, LaSalle JM, Van den Veyver IB (2006). Expression profiling of clonal lymphocyte cell cultures from Rett syndrome patients. BMC Med. Genet..

[65] Kerr D, Krishnan C, Pucak ML, Carmen J (2005). The immune system and neuropsychiatric diseases. Int. Rev. Psychiatry.

[66] Khandaker GM (2015). Inflammation and immunity in schizophrenia: implications for pathophysiology and treatment. Lancet Psychiatry.

[67] Lengauer C, Issa J-P (1998). The role of epigenetics in cancer. Mol. Med. Today.

[68] Paciorkowski AR (2013). MEF2C Haploinsufficiency features consistent hyperkinesis, variable epilepsy, and has a role in dorsal and ventral neuronal developmental pathways. Neurogenetics.

[69] Ghosh A, Carnahan J, Greenberg ME (1994). Requirement for BDNF in activity-dependent survival of cortical neurons. Science (80-).

